# Phosphate as an adjunct to calcium in promoting coronary vascular calcification in chronic inflammatory states

**DOI:** 10.7554/eLife.91808

**Published:** 2024-06-12

**Authors:** Gordon L Klein

**Affiliations:** 1 Department of Orthopaedic Surgery and Rehabilitation, University of Texas Medical Branch Galveston United States; Icahn School of Medicine at Mount Sinai United States; University of Michigan–Ann Arbor United States

**Keywords:** calcium, phosphate, calcium-sensing receptor, inflammation, atherosclerosis, bone

## Abstract

Bone releases calcium and phosphate in response to pro-inflammatory cytokine-mediated inflammation. The body develops impaired urinary excretion of phosphate with age and chronic inflammation given the reduction of the kidney protein Klotho, which is essential to phosphate excretion. Phosphate may also play a role in the development of the resistance of the parathyroid calcium-sensing receptor (CaSR) to circulating calcium thus contributing to calcium retention in the circulation. Phosphate can contribute to vascular smooth muscle dedifferentiation with manifestation of osteoblastogenesis and ultimately endovascular calcium phosphate precipitation. Thus phosphate, along with calcium, contributes to the calcification and inflammation of atherosclerotic plaques and the origin of these elements is likely the bone, which serves as storage for the majority of the body’s supply of extracellular calcium and phosphate. Early cardiac evaluation of patients with chronic inflammation and attempts at up-regulating the parathyroid CaSR with calcimimetics or introducing earlier anti-resorptive treatment with bone active pharmacologic agents may serve to delay onset or reduce the quantity of atherosclerotic plaque calcification in these patients.

## Introduction: Chronic inflammation and bone loss

A recent review of chronic inflammatory conditions involving bone loss focused on four conditions with documented increase in risk for atherosclerotic cardiovascular disease: post-menopausal osteoporosis, spinal cord injury, osteoarthritis and rheumatoid arthritis (Klein, 2022). These conditions all involve resorptive bone loss and all have epidemiologic evidence of increased risk for atherosclerotic heart disease compared to individuals who do not suffer from these conditions. While we have constructed a model to trace a path of calcium from resorbing bone to coronary arteries, that pathway for phosphate is less clear. However, we do know that 85% of the body’s phosphate is stored in bone, and in inflammation the bone liberates phosphate along with calcium. Moreover, calcifications in the coronaries, as well as the carotids, have been identified as calcium phosphate hydroxyapatite (Saba et al., 2022).

The purpose of this review is to describe (a) the epidemiologic evidence that resorptive bone loss and coronary calcium accumulation may correlate with each other, (b) why phosphate and calcium may be supersaturated in the blood, (c) that phosphate may be retained by the body, that is, not excreted in the urine, during chronic inflammation, (d) the evidence that supersaturated phosphate can work together with calcium toward the development of precipitates as well as actual bone in the coronary vessels, the significance of serum phosphate levels in coronary vascular calcium phosphate precipitation, (e)experimental evidence for a role of phosphate in vascular precipitation of calcium phosphate, and (f) investigations that could be undertaken to ascertain whether preventative intervention can reduce the extent of coronary artery calcification, thereby reducing morbidity and mortality from ischemic heart disease.

### Epidemiology

Campos-Obando et al., 2015 and Lee et al., 2016a found correlations between the amount of bone loss and coronary artery calcium in women with post-menopausal osteoporosis. With regard to spinal cord injury, Qin et al., 2010 describe the resorption of bone and prevalence of inflammation occurring over the same time period, and both Bauman and Spungen, 2008 and Peterson et al., 2021 noted the increased incidence of cardiovascular disease in spinal cord injured patients compared to matched individuals for age, gender, and ethnicity. In osteoarthritis, meta-analyses by Wang et al., 2016. Hall et al., 2016 and Macêdo et al., 2022 demonstrated increased odds ratio of cardiovascular disease compared to controls not suffering from the condition, especially in those with hip and knee osteoarthritis (Macêdo et al., 2022). For rheumatoid arthritis, (Liao, 2017) noted an increased incidence of cardiovascular disease of 1.5–2 times that in age and sex-matched individuals who did not suffer from the condition, while Karpouzas et al., 2020 observed that coronary artery calcium score and plaque progression were greater in older patients with increased inflammation compared to patients who did not demonstrate disease progression.

Moreover, epidemiologic studies of cardiovascular mortality in rheumatoid arthritis patients (Wolfe et al., 2013) who were taking bisphosphonates demonstrated a reduction compared to those who were not taking the anti-resorptive medication. In addition, all-cause mortality of intensive care patients who had been taking bisphosphonates prior to hospital admission was significantly lower than that of patients in the same unit who had not been receiving these medications (Lee et al., 2016b). Specifically relevant to phosphate was another study by Campos‐Obando et al., 2022, who performed a Mendelian randomization study that associated serum phosphate concentration with coronary artery calcification.

These studies point to a possible relationship between bone resorption and coronary artery accumulation of calcium and phosphate in chronic inflammatory conditions. In a recently published review of bone calcium as a factor in the pathogenesis of atherosclerotic heart disease in chronic inflammation (Klein, 2022), evidence was described suggesting that the parathyroid calcium-sensing receptor (CaSR) became less sensitive to circulating calcium in adults compared to children and that reduced sensitivity led to increased parathyroid hormone production by the parathyroid chief cells with consequent increased calcium retention following inflammatory bone resorption.

Inasmuch as phosphate as well as calcium is resorbed from bone in chronic inflammation, the purpose of this review is to examine how phosphate released by bone in chronic inflammatory states may be retained by the body and its role(s) in the pathogenesis of coronary artery disease in these patients.

### Why might bone be a source of phosphate and why may phosphate be supersaturated in the blood?

In addition to the above-mentioned note that bone stores 85% of the body’s phosphate, Villa-Bellosta et al., 2011 and Kuro-o, 2021 state that in vertebrates calcium and phosphate circulate at a concentration close to the solubility constant of calcium phosphate in order for their stronger bones to support body weight on land and that slight increases in the circulating concentrations of phosphate could result in precipitation. Causes of slight increases in circulating phosphate are most commonly unregulated dietary intake of phosphate (Rubio-Aliaga and Krapf, 2022) and likely inflammatory bone resorption. While it is not clear exactly how much phosphate is liberated by bone during chronic inflammation, and the quantity may vary with the intensity of the inflammatory response, the amount released remains likely to increase serum phosphate concentration, at least transiently. Therefore, the concentration of phosphate in the circulation at any one point in time may not be indicative of the phosphate that precipitates in the blood vessels as calcium phosphate precipitation could lower the circulating phosphate concentration.

### How is phosphate excreted from the body?

Kuro-o, 2021 described a deficiency of the Klotho gene, expressed in the distal convoluted tubule of the kidney, which is associated with aging and inflammation. Transmembrane Klotho protein binds with specific isoforms of the fibroblast growth factor (FGF) receptor, namely FGF-1c,–3c, and –4. While FGF-23 cannot bind to these receptors, it can bind tightly to the FGFR-Klotho complex so that Klotho serves as a co-receptor for FGF-23. The FGF-23-Klotho complex protects the kidney from phosphate overload, inhibiting renal tubular 1α-hydroxylase, thus decreasing 1,25-dihydroxyvitamin D production and consequent intestinal phosphate absorption while inhibiting the renal tubular sodium-phosphate reabsorptive transporter NaPi-IIa in the apical plasma membrane of the proximal tubule (Quarles, 2012). As Klotho is reported decreased with inflammation and aging (Quarles, 2012) and as the FGF-23-Klotho pathway is a major mechanism of urinary phosphate excretion, it is likely that urinary phosphate excretion is reduced in chronic inflammation. Inflammation has been shown to reduce Klotho in rats with lipopolysaccharide-induced acute inflammation but not in those with hypovolemic distress (Ohyama et al., 1998). In addition, Klotho expression has been reported to be reduced in mice with inflammatory bowel disease, while the use of anti-tumor necrosis factor α (TNFα) resulted in attenuation of the inflammation and reversal of the Klotho suppression ( Thurston et al., 2010). Moreno et al., 2011 demonstrated in renal tubular cells that TNFα and TWEAK cytokines reduced Klotho gene transcription and that the transcription factor nuclear factor κB (NF κB) Rel A was required for suppression of Klotho gene transcription. In addition, Klotho has anti-inflammatory properties. Thus Liu et al., 2011 have shown that Klotho suppresses retinoic acid-inducible gene (RIG-I), which mediates senescence-associated inflammation via NFκB and interferon regulatory factor (IRF). During kidney aging, Klotho decreases and RIG-I and IL-6 increase. Therefore, if less phosphate is excreted during chronic inflammation, more phosphate is either retained in the circulation or precipitated.

How Does Phosphate Work Together with Calcium to Create Vascular Calcification and What is the Signifiance of Serum Phosphate Concentration in Evaluating Risk for Precipitation? (New section in Bold type like previous section head!).

To this point, we have identified bone as a source of phosphate and that resorption can lead to the liberation of bone phosphate as well as calcium. Moreover, in the presence of chronic inflammation, while bone is resorbed and phosphate enters the circulation, its urinary excretion may be reduced leading to either increased circulating levels of phosphate or actual phosphate precipitation. The current section will discuss how, once in the circulation, phosphate and calcium can interact in ways that lead to vascular precipitation.

A recent study by Centeno et al., 2019 identified phosphate anion binding sites on the parathyroid calcium-sensing receptor (CaSR). This phosphate binding changes the conformation of the CaSR so that it cannot up-regulate in the presence of circulating calcium. Therefore, the CaSR cannot prevent the parathyroid chief cell from producing and secreting parathyroid hormone (PTH). Persistence of PTH even in the presence of higher circulating calcium following inflammation-induced bone resorption allows the kidney to retain calcium in the circulation and thus to exert its pro-inflammatory effects as previously discussed (Klein, 2022). Inasmuch as children retain more phosphate to mineralize growing bone, when these children reach their maximum height and phosphate is no longer required to mineralize growing bones, one can speculate that some of that phosphate can now be free to bind to the parathyroid CaSR. While it is not presently clear that phosphate binding to the CaSR is the mechanism by which the CaSR is inhibited in adults, it remains plausible. Cirillo et al., 2008 noted that in adults ranging in age from 18 to 97 years, both serum levels of phosphate and renal tubular phosphate reabsorption (TmP/GFR) declined with age. Thus, as Villa-Bellosta et al., 2011 and Kuro-o, 2021 state, if calcium and phosphate are super-saturated in the circulation and continue to enter the blood through bone resorption, younger adults would be more likely to be hyperphosphatemic than older adults (Cirillo et al., 2008), increasing the likelihood that the phosphate binding sites on the parathyroid CaSR may be more occupied, thus resulting in an increase in PTH secretion and consequent calcium retention. While serum phosphate and tubular phosphate reabsorption decline with age, it is also possible that calcification begins in the arteries when patients are younger and more phosphate may be excreted by older adults as muscle mass, which utilizes phosphate for ATP production, declines with age.

The consequence of this loss of CaSR responsiveness is the inability of the body to dump excess calcium in the urine, thus prolonging its circulatory effect and its ability to sustain the inflammatory response or to precipitate in the blood vessels. As the circulating calcium passes through smaller vessels, such as the coronary arteries, it can initiate vascular endothelial inflammation via its stimulation of chemokine production (Klein, 2022) and can interact with the vascular endothelial CaSR (Klein et al., 2008) to increase arterial tone and vascular narrowing (Schepelmann et al., 2016).

In addition to its potential role in binding to the parathyroid CaSR, phosphate and calcium in the circulation can undergo precipitation, although the body has another mechanism to attempt to prevent that, adsorption of amorphous calcium and phosphate by the hepatic protein fetuin A, to form calciprotein monomers, or primary calciprotein (CPP; Kuro-o, 2021). These primary CPPs can spontaneously undergo a phase transition from amorphous to crystalline CPPs, otherwise known as secondary CPPs. These secondary CPPs may function as a vehicle that carries calcium and phosphate back to bone (Herrmann et al., 2012; Akiyama et al., 2020).When the capacities of secondary CPPs are exceeded, they can induce FGF-23 expression and secretion in osteoblasts and osteocytes (Akiyama et al., 2020). However, secondary CPPs can also induce calcification of cultured vascular smooth muscle cells. Moreover, clinical studies have reported that serum CPP concentration correlates with parameters of coronary artery calcification scores, vascular stiffness, and inflammation in the form of hs-CRP in patients with chronic kidney disease (Hamano et al., 2012; Smith et al., 2012).

### What is the experimental evidence of phosphate effect on vascular calcification?

An in vitro study of human aortic smooth muscle grown in media with differing phosphate concentrations by Jono et al., 2000 showed that human aortic smooth muscle cells did not mineralize in media with an inorganic phosphate (Pi) concentration of 1.4 mmol/l, normal, but mineralized in a dose-dependent fashion. The presence of Pi >1.4 mmol/l also increased osteoblastic differentiation markers of osteocalcin and runx2 (cbfa1) with the phosphate effects mediated by the sodium-dependent phosphate co-transporter Pit-1. In a study of rats by Yamada et al., 2014, normal rats fed a phosphate diet of 1.2% (high phosphate) had no increase in serum or tissue levels of TNF alpha. However, rats with chronic kidney disease had a dietary Pi concentration-dependent rise in serum and tissue levels of TNF alpha and tissue levels of oxidative stress markers. Cultured vascular smooth muscle cells with high medium Pi directly increased expression of TNF alpha prior to observing an increase in osteochondrogenic markers. Interestingly, in the rats, multivariate analysis showed serum TNF alpha correlated with aortic calcium after adjusting for creatinine clearance, suggesting that the association was independent of kidney function, with correlation coefficient of 0.405, p<0.05. Steitz et al., 2001 showed that vascular smooth muscle cells exposed to high amounts of phosphate as would occur in chronic kidney disease lose expression of smooth muscle contractile proteins SM 22 alpha and SM alpha actin and express bone markers runx2, osteopontin, osteocalcin, and alkaline phosphatase.

Vascular smooth muscle cells produce bodies known as matrix vesicles which serve as mineral nucleation sites and are responsible for the initial deposition of calcium and phosphate in blood vessels. Matrix vesicles originate from dedifferentiated or calcifying smooth muscle cells. High concentrations of calcium in the cytosol of these cells promote translocation of annexin 6 to the smooth muscle cell plasma membrane. This stimulates the release of vesicles with a Ca^2+^-Pi-phosphatidyl serine as the nucleation core (Kapustin and Shanahan, 2012). Matrix vesicles may contain the bone protein, Matrix GLA protein, or MGP. This protein is a calcification inhibitor. Phosphate has not been shown to affect the amount of MGP that enters a matrix vesicle (Chen et al., 2018) and the role of MGP in vascular calcification is still unclear.

## What is the clinical relevance ot these findings?

Thus, phosphate plays a role in vascular calcification with chronic inflammation. It is liberated from bone during resorption, along with calcium. It may contribute to the development of non-competitive inhibition of the parathyroid calcium-sensing receptor, thus facilitating the retention in the circulation of resorbed calcium in the circulation allowing for the inflammatory effects of calcium in the vessels as well as the increase in vascular tone due to interaction with the endothelial calcium sensing receptor (Klein, 2022). Phosphate may also stimulate the osteogenic differentiation of vascular smooth muscle and contribute to the development of the hydroxyapatite crystal formation in the coronary arteries. Serum phosphate concentration, if elevated, may be a risk factor for calcium phosphate vascular precipitation. However, normal circulating phosphate concentration may not preclude occurrence of precipitation given what may be the transience of serum phosphate elevations.

## What can be done to prevent or attenuate the development of atherosclerotic changes with chronic inflammation?

Two approaches are suggested. The first is the trial of calcimimetics. Here, the attempt is not to diminish the resorption of bone and consequent liberation of phosphate but to attempt to restore the ability of the parathyroid CaSR to upregulate in response to high circulating calcium in order to reduce PTH secretion by the gland, allowing for increased urinary excretion of calcium. Drugs such as cinacalcet have slowed progression of vascular calcification and atherosclerosis in rats and mice with chronic kidney disease, as summarized by Drueke (Drüeke, 2013), while clinical trials have been few and inconclusive to date. However, further trials would be indicated inasmuch as the clinical trials were conducted in patients with chronic kidney disease stage 5D on dialysis, and even there, in the EVOLVE study, there was a slight, although statistically not significant, reduction in the number of cardiovascular events, 7%, in the cinacalcet group and the results from animal studies have been promising. Clinical studies at an earlier disease stage as well as in other chronic inflammatory conditions such as osteoarthritis and rheumatoid arthritis, spinal cord injury, and post-menopausal osteoporosis could also yield promising results.

The second approach might be for earlier cardiovascular evaluation of patients with chronic inflammatory diseases and earlier initiation of anti-resorptive therapy. One potentially promising approach might be the examination of bone density scans of the lateral lumbar spine for the presence of abdominal aortic calcifications (Schousboe et al., 2017), especially since they may correlate with the presence of coronary artery calcifications (Dalla Via et al., 2023).

While Kim et al., 2015 performed a meta-analysis of 58 studies on the effects of bisphosphonates on the risk of cardiovascular disease and found no significant preventative effect of bisphosphonates, it must be noted that the studies analyzed entailed the use of bisphosphonates initiated at a time when they are indicated by either bone density T scores or FRAX scores. By that time, if coronary artery calcification is well advanced, the use of anti-resorptive agents may not be effective in preventing or ameliorating cardiovascular calcifications. If screening lateral lumbar spine bone density scans for abdominal aortic calcifications is effective in earlier detection of vascular calcification in any of the chronic inflammatory conditions studied for higher risk of cardiovascular disease, it is possible that criteria for initiation of anti-resorptive therapy could be re-evaluated.

Finally, a meta-analysis by Ruospo et al., 2018 examined the efficacy of the use of phosphate binders in chronic kidney disease including dialysis on all-cause mortality without any evidence of any drug class significantly lowering mortality or cardiovascular events compared to placebo. However, the mean duration of at least 77 of the 104 trials was 6 months, which may not have been adequate to detect any clinical effect.

**Figure 1.**
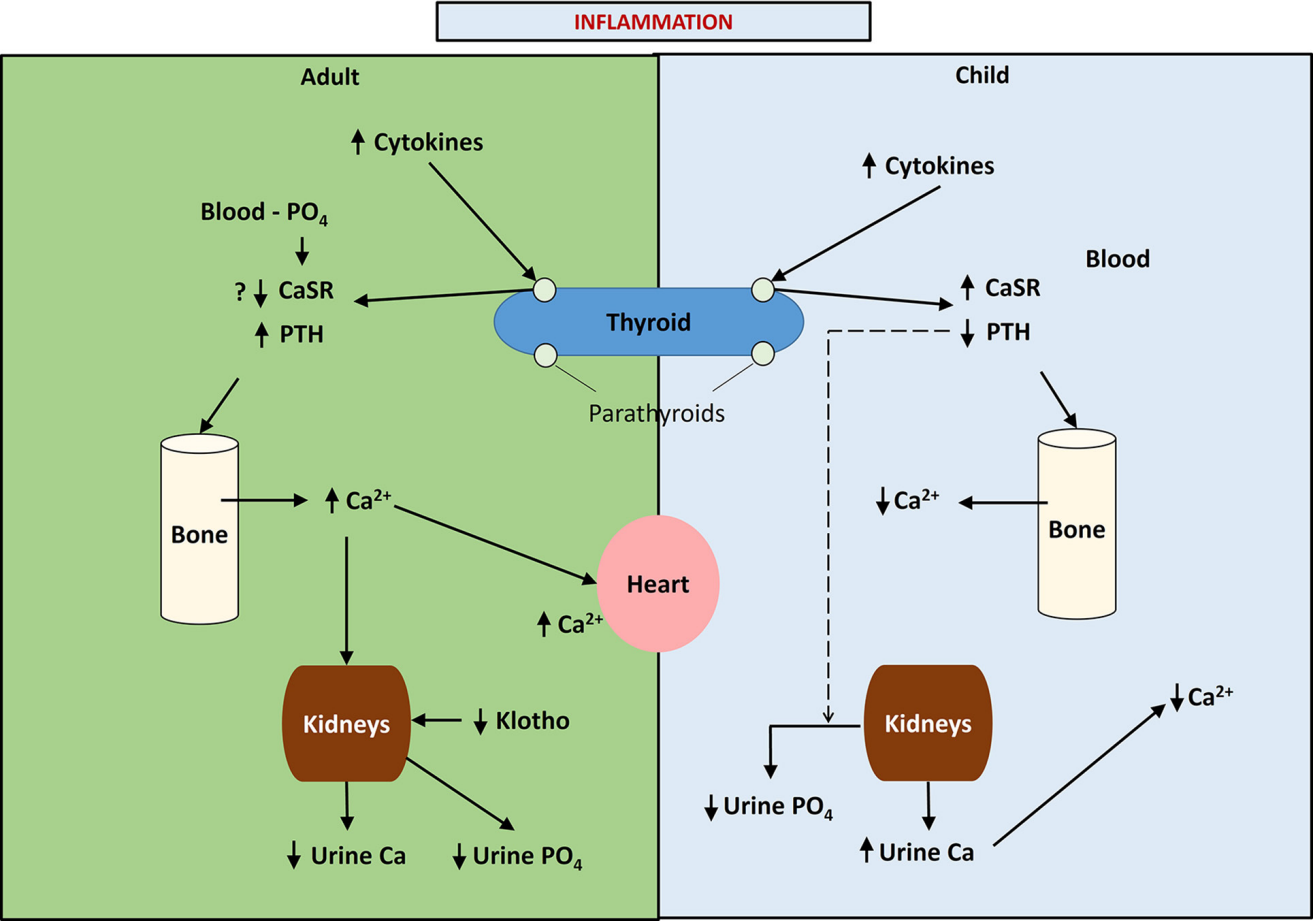
This figure provides a schematic diagram of the differences in calcium handling in previously healthy children and adults following acute burn injury and the putative role of phosphate in adults and children. Note that in children, pro-inflammatory cytokines up-regulate the parathyroid calcium-sensing receptor (CaSR) leading to hypocalcemic hypoparathyroidism and increased urinary calcium excretion, while in adults the up-regulation of the parathyroid CaSR response to pro-inflammatory cytokines is inhibited, possibly due to phosphate binding to the parathyroid CaSR leading to normo-or mildly hypercalcemic hyperparathyroidism and a reduction in urinary calcium excretion.

An outline of the differences between calcium, phosphate, and PTH handling between children and adults is illustrated in Figure 1.

Thus, evidence has been presented that calcium and phosphate from bone resorption can be the sources of the calcifications that precipitate in coronary vessels in the pathogenesis of atherosclerosis. This conclusion is also supported by the fact that bone contains the single largest body stores of both extracellular calcium and extracellular phosphate. Experimental evidence suggesting that extracellular phosphate may inhibit the calcium sensing receptor up-regulation in response to circulating calcium, that Klotho declines with age and inflammation likely reducing renal phosphate excretion, and pathologic evidence of calcium phosphate precipitates in atherosclerotic plaques all point to a role of phosphate in the pathogenesis of cardiovascular disease in association with calcium retention.

## Unanswered questions

Clearly, the scenario presented here does not take into account other mechanisms that may also be involved in the pathogenesis of atherosclerotic plaque calcification and which remain to be identified. However, the above mechanisms which have been discussed can serve as a way of understanding how the body can create pathways from bone resorption to atherosclerotic plaque calcifications. Furthermore, this scenario identifies potential therapeutic targets which can be investigated to determine new ways of preventing or delaying onset of cardiovascular disease with chronic inflammation.

Among the challenges remaining are finding a direct method of examining circulating phosphate kinetics in the body, better understanding the conditions favoring calcium phosphate precipitation in blood vessels, elucidating the roles of matrix GLA protein, pyrophosphate, and osteopontin in inhibition of calcium phosphate precipitation, and sorting the genetic mechanisms that link hyperphosphatemia, aging and chronic inflammation. Increasing our understanding of these factors may provide more and better therapeutic targets to prevent or attenuate the pathologic effects of vascular calcification.
